# Distribution of hepatitis B virus subgenotype F2a in São Paulo, Brazil

**DOI:** 10.1186/1756-0500-6-423

**Published:** 2013-10-21

**Authors:** Mónica V Alvarado-Mora, Livia S Botelho-Lima, Rubia A Santana, Roberta Sitnik, Paulo Abrão Ferreira, Francisco do Amaral Mello, Cristovão P Mangueira, Flair J Carrilho, João R Rebello Pinho

**Affiliations:** 1 Laboratory of Tropical Gastroenterology and Hepatology, Department of Gastroenterology, School of Medicine, University of São Paulo, São Paulo, SP, Brazil; 2 Albert Einstein Diagnostic Medicine, São Paulo, SP, Brazil; 3 Federal University of São Paulo - UNIFESP, São Paulo, SP, Brazil; 4 Oswaldo Cruz Foundation, FIOCRUZ, Rio de Janeiro, Brazil

**Keywords:** Hepatitis B, Brazil, Bayesian analysis, Subgenotype F2a

## Abstract

**Background:**

HBV genotype F is primarily found in indigenous populations from South America and is classified in four subgenotypes (F1 to F4). Subgenotype F2a is the most common in Brazil among genotype F cases. The aim of this study was to characterize HBV genotype F2a circulating in 16 patients from São Paulo, Brazil. Samples were collected between 2006 and 2012 and sent to Hospital Israelita Albert Einstein. A fragment of 1306 bp partially comprising HBsAg and DNA polymerase coding regions was amplified and sequenced. Viral sequences were genotyped by phylogenetic analysis using reference sequences from GenBank (n=198), including 80 classified as subgenotype F2a. Bayesian Markov chain Monte Carlo simulation implemented in BEAST v.1.5.4 was applied to obtain the best possible estimates using the model of nucleotide substitutions GTR+G+I.

**Findings:**

It were identified three groups of sequences of subgenotype F2a: 1) 10 sequences from São Paulo state; 2) 3 sequences from Rio de Janeiro and one from São Paulo states; 3) 8 sequences from the West Amazon Basin.

**Conclusions:**

These results showing for the first time the distribution of F2a subgenotype in Brazil. The spreading and the dynamic of subgenotype F2a in Brazil requires the study of a higher number of samples from different regions as it is unfold in almost all Brazilian populations studied so far. We cannot infer with certainty the origin of these different groups due to the lack of available sequences. Nevertheless, our data suggest that the common origin of these groups probably occurred a long time ago.

## Findings

Despite the availability of an effective vaccine, more than 350 million people worldwide are chronically infected with hepatitis B virus (HBV), and many people have developed severe liver diseases, such as cirrhosis and hepatocellular carcinoma (HCC) [1]. HBV has a partially double-stranded DNA genome composed of approximately 3.2 kb, with four overlapping open reading frames (ORF): preS/S, precore/core, X and polymerase) [2]. As a result of its unusual mechanism of replication by reverse transcription and the lack of proofreading activity of this enzyme, it displays a highly heterogeneous sequence diversity.

Nine HBV genotypes (HBV/A to HBV/I) and a controversial HBV/J have been reported based on the differences in full-length genome sequences [3]. Genotype A (A1 to A7) is highly prevalent in sub-Saharan Africa (subgenotype A1), Northern Europe (subgenotype A2), and Western Africa (subgenotype A3). Furthermore, both A4 and potential A5 strains were found in sub-Saharan Africa (Mali, Gambia and Nigeria) [4,5]. Recently, A5 was also found in African descendants in Haiti [6]. Proposed subgenotype A6 include strains from African–Belgian patients originating from Congo and Rwanda [7].

HBV/B and HBV/C genotypes are common in Asia. Currently, genotype B is divided in subgenotypes B1 to B6. Among them, B1 is found mainly in Japan, B2–5 in East Asia, and B6 in indigenous populations living in the Arctic, such as Alaska, Northern Canada and Greenland [8]. HBV genotype C (C1 to C5) is found throughout the eastern and southeastern portions of Asia and the Pacific islands (Micronesia, Melanesia, and Polynesia), as well as in immigrants from these areas in the United States, Europe, Australia, New Zealand [9] and Brazil [10]. HBV/D with subtypes D1–D5 is prevalent in the Mediterranean region expanding through South Asia up to India [8]. HBV/E is restricted to West Africa [11] but some studies reported the presence of this genotype in South America [12] and India [13]. This genotype is characterized by a low genetic diversity among strains from throughout the world [14]. Genotype G has been reported in USA and France [15]. HBV/F and HBV/H genotypes are found in the Americas, with genotype F described from Alaska to Argentina, while genotype H has been mainly described in Central America and Mexico [16].

HBV genotypes F and H are considered indigenous to the American continent [15,17,18]. An HBV strain isolated from woolly monkey (a New World monkey) is closer to genotype F but with a noteworthy phylogenic distance from other strains of HBV [19]. Several strains of genotype F have been isolated in different countries in America, especially among Amerindian population and four subgenotypes have been described with genetic divergence among 4.3–6.1% [20].

HBV genotypes and subgenotypes have distinct geographical distribution and it is currently discussed if they are associated with different prognosis considering response to antiviral treatment and the severity of liver disease in different populations. Nevertheless, global human migrations affect the pattern of HBV genotypes distribution, introducing genotypes differing from those found in the original inhabitants.

HBV genotypes A, D and F are the most frequent in São Paulo [21], but there are not any published data on subgenotypes found there. It was previously reports that subgenotype F2 is the most frequent in Brazil [22], particularly subgenotype F2a, as latter subclassified by Devesa et al. [20]. In this study, we compare HBV subgenotype F2a sequences recently obtained in São Paulo from chronic hepatitis B patients with other sequences previously described around the world with the aim to determine the origin of subgenotype F2a circulating in São Paulo, Brazil.

Nineteen samples from chronically infected patients (seventeen male and two female) with HBV subgenotype F2a were obtained between 2006 to 2012 at Albert Einstein Diagnostic Medicine. The study was approved by the research Ethics committee of Albert Einstein Hospital (CEP/Einstein No 10/1293) and an informed consent for participation in the study was obtained from each patient.

These samples were initially subgenotyped using a system developed in the Clinical Laboratory [23] and were then selected for a longer fragment (1306 bp) amplification using other previously described primers [18].

For viral DNA extraction from 200 μl serum, it was used a commercial available kit (QIAamp mini, QIAGEN, Germany), according to the manufacturer’s instructions. Precipitated DNA was resuspended in 200 μl of elution buffer and stored at -20°C until use. To avoid false-positive results, strict procedures proposed for nucleic acid amplification diagnostic techniques were followed [24]. The amplified DNA was purified using ChargeSwitch® PCR Clean-Up kit (Life Technologies, São Paulo, Brazil). Sequencing was performed in an ABI 3500 Genetic Analyzer (Life Technologies, Foster City, CA, USA) using dideoxynucleoside triphosphates (ddNTPs) containing fluorescent markers (Big Dye® Terminator v3.1 Cycle Sequencing Ready Reaction kit – Life Technologies, Foster City, CA, USA). The quality of each electropherogram was evaluated using the Phred-Phrap software [25] and consensus sequences were obtained by alignment of both sequenced strands (sense and antisense) using CAP3 software available at the web page Eletropherogram quality analysis Phred (http://www.biomol.unb.br/phph/).

HBV sequences were aligned with other previously reported genome sequences (n = 198) using ClustalX software [26] and was edited with the SE-AL software (available from: tree.bio.ed.ac.uk/software/seal/). The Bayesian Markov chain Monte Carlo simulation implemented in BEAST v.1.4.8 [27] was applied to obtain the best possible estimates using both the relaxed uncorrelated log_normal_ and exponential molecular clock models and using the model of nucleotide substitution (general time-reversible + gamma + proportion invariant). The molecular clock that best fits the data was chosen by Bayes factor comparison. After 20 million generations, the maximum clade credibility (MCC) tree was obtained by summarizing 20,000 substitution trees and was then modified using a 10% burn-in using Tree Annotator v.1.5.3 [27]. Phylogenetic trees were visualized and midpoint rooted in FigTree v1.2.2 (tree.bio.ed.ac.uk/software/figtree/).

**Figure 1:**
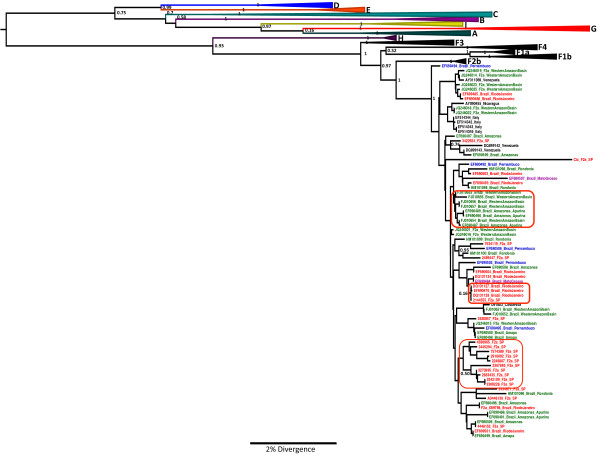
**The Maximum Clade Credibility (MCC) tree was estimated by a Bayesian analysis of 198 S/POL sequences of hepatitis B virus strains.** The posterior probabilities of the key nodes are shown above the respective nodes. The HBV/F2a samples obtained from Sao Paulo (n=19) were analyzed together with other strains from around the world. Brazilian sequences from North Region (green), Northwest Region (blue), Southeast Region (red) and Center West Region (purple) were highlighted in tree. The clusters containing the strains of other HBV genotypes collapsed.

**Figure 2:**
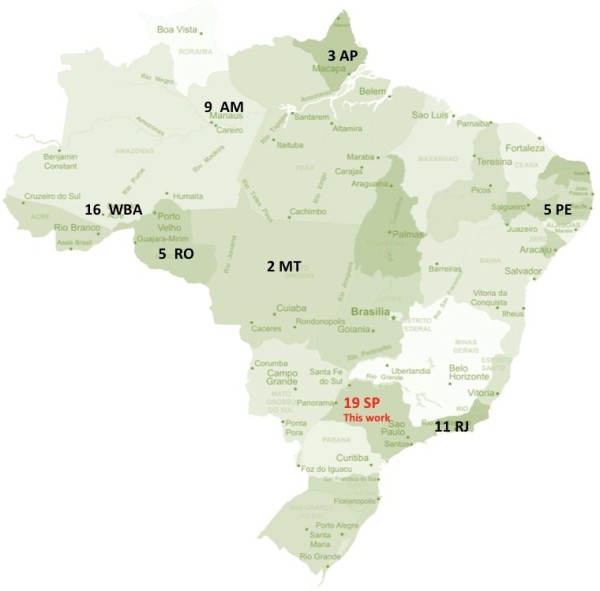
Geographic location of HBV sequences from Brazil: North Region (3 AP - Amapá State, 5 RO - Rondônia State, 26 AM - Amazonas State, including 5 from Manaus, the capital city, 5 from the Apurinãs people and 16 WAB from Western Amazon Basin (Apurinãs and WAB samples were obtained from riverside communities living near Purus River); Northwest Region (5 PE -Pernambuco State); Southeast Region (11 RJ - Rio de Janeiro State and 19 SP - São Paulo State) and Center West Region (2 MT - Mato Grosso State).

A phylogenetic tree constructed with the 1,306 bp sequences partially comprising HBsAg and DNA polymerase coding regions (S/POL) (n = 198) is shown in Figure 1, including the 19 new sequences obtained by our group from São Paulo state. Bayesian analyses were performed to try to determine the grouping of the genotype F2a sequences available. The complete dataset included 71 sequences from Brazil: North Region (3 AP - Amapá State, 5 RO - Rondônia State, 26 AM - Amazonas State, including 5 from Manaus, the capital city, 5 from the Apurinãs people and 16 WAB from Western Amazon Basin (Apurinãs and WAB samples were obtained from riverside communities living near Purus River); Northwest Region (5 PE -Pernambuco State); Southeast Region (11 RJ - Rio de Janeiro State and 19 SP - São Paulo State) and Center West Region (2 MT - Mato Grosso State) (Figure 2).

The phylogenetic tree showed three groups containing sequences from the same state that grouped with a high a posteriori probability: a branch of sequences from the WAB, including Apurinãs samples, all from the North Region and two branches of sequences from the Southeastrn regions, one containing mostly sequences from Rio de Janeiro State and another with 10 sequences from São Paulo State. The other sequences did not show a pattern organized by Brazilian states or regions.

Currently, subgenotype F2a has been mostly found in Venezuela and it has been considered as originating from the Amazon region, this subgenotype has been spreading through the different entries in different regions so diverse.

We used all of genotype F sequences available in the GenBank and applied a coalescent-based approach. Ten HBV F2a sequences of patients from São Paulo were grouped in one cluster. It was not found specific relation or similar characteristics among these 10 patients when compared with the other nine sequences from São Paulo.

Since most of the subgenotype F2a sequences are from Brazil and it is not reported from which state each sequence was obtained, it was not possible to infer its routes of spread in this work. The spreading and the dynamic of subgenotype F2a in Brazil requires the study of a higher number of samples from different regions as it is unfold in almost all Brazilian populations studied so far. However, these results showing for the first time the distribution of F2a subgenotype in Brazil.

In conclusion, we identified three groups of sequences of subgenotype F2a: 1) 10 sequences from São Paulo state; 2) 3 sequences from f Rio de Janeiro and one from São Paulo states; 3) 8 sequences from the West Amazon Basin. We cannot infer with certainty the origin of these different groups due to the lack of available sequences. Nevertheless, our data suggest that the common origin of these groups probably occurred a long time ago. In this sense, more studies should be carried out with HBV infected populations in our country, particularly with indigenous populations in other regions of our country, particularly in the regions where the indigenous populations were common at the time of the beginning of Brazil’s colonization by European populations.

## Competing interests

The authors declared that they have no competing interests.

## Authors’ contributions

MVAM participated in the design of the study and drafted the manuscript, conducted the sequencing process and phylogenetic analysis. LSBL and RAS participated in the PCR amplification and sequencing process. RS, PAF, CPM and FJC participated in the design of the study. FAM participated with some previously published data of Brazilian sequences. JRRP participated in the design of the study and the elaboration of the manuscript. All authors read and approved the final manuscript.
